# How does COVID-19 affect people’s ability to smell

**Published:** 2022-04

**MARCH 23, 2022** - A decreased or altered sense of smell—called olfactory dysfunction—is a common symptom experienced by individuals with COVID-19. As described in an article published in The Laryngoscope, researchers recently searched the medical literature for studies reporting changes in olfactory structures detected through imaging tests of patients with COVID-19.

The prevalence of an olfactory cleft abnormality was nearly 16-fold higher in patients with COVID-19 and olfactory dysfunction (63%) compared with controls (4%). The olfactory clefts provide a crucial channel for airborne molecules to reach sensory olfactory neurons that connect to the brain to enable a person to perceive smells.

“Before this study, most scientists thought that the loss of smell in COVID-19 was mainly due to inflammation and damage to the olfactory nerves. Now, we have compiled evidence from medical imaging that COVID-19 loss of smell is also due to swelling and blockage of the passages in the nose that conduct smells,” said senior author Neville Wei Yang Teo, MRCS, MMed, of Singapore General Hospital.

“We think this is good news for patients who want to recover their sense of smell, since these blockages are expected to resolve with time, while nerve damage in comparison would likely be more difficult to recover from,” added lead author Claire Jing-Wen Tan, of the National University of Singapore. “These findings may not fully account for those who suffer from prolonged olfactory dysfunction, however, and further studies that evaluate patients in this group may provide more information.”

***Link to Study:** https://onlinelibrary.wiley.com/doi/10.1002/lary.30078*

***Full citation:** “Neuroradiological Basis of COVID-19 Olfactory Dysfunction: A Systematic Review and Meta-Analysis” Claire Jing-Wen Tan, Benjamin Kye Jyn Tan, Xin Yan Tan, Hui Ting Liu, Chong Boon Teo, Anna See, Shuhui Xu, Song Tar Toh, Si Wei Kheok, Tze Choong Charn, Neville Wei Yang Teo, Laryngoscope; Published Online: 22 March 2022 (DOI:10.1002/lary.30078).*

*Copyright © 2019 The Cochrane Collaboration. Published by John Wiley & Sons, Ltd., reproduced with permission.*

